# Mr. Gibbon's Reply to Dr. Yeats

**Published:** 1814-01

**Authors:** James Gibbon

**Affiliations:** Bedford


Your very obedient Servant,

J. S. GRIMSHAW,

*Vicar of Bidenham, Beds.*

*Bedford, Dec.* 9, 1813.

---

*To the Editor of the Medical and Physical Journal.*

SIR,

I MUST again trespass upon your indulgence in soliciting a page of the Journal, for the purpose of remarking upon the letter of Dr. Yeats which appeared in the last Number.

The question at issue is simply, whether Ann Foulkes was guilty of deception or not. I believe her to have acted deceptiously. To substantiate that belief, I refer to the case which which I have published; and if Dr. Yeats cannot dispute or controvert the statement which I have publicly given, it is incumbent upon him to acknowledge the imposture, as no medical man can entertain a different opinion upon the subject, unless he can disprove the circumstances of the case as brought forward by me.

I expressed surprise at the unfairness of Dr. Yeats's conduct in reference to me about this reputed case of urinous vomiting. I must repeat my surprise at his last letter, which imperiously calls upon me to offer some explanations, and likewise act the part of a prompter to the doctor's memory:

> Sed tali auxilio ac defensoribus istis
> Tempus multum eget.

Can Dr. Yeats have forgotten that I was twice present at a meeting of *all* those medical gentlemen he alludes to, previously to their visiting Ann Foulkes, when he himself was likewise of the party?

I next find that Dr. Y. reverts to the subject of this medical meeting or consultation as being attended with ungracious appearances. Who would believe that he most fully acquiesced in the positive declaration of five medical men that their meeting at Bedford was purely accidental? No man could appear to be more satisfied with the explanation which I gave to him, respecting this meeting, than he did. I must beg leave to refer him to his own assertion in the 45th page of the Medical and Physical Journal for July 1813, where he expressly declares it was a casual meeting. So much so was it, in reality, that neither Dr. Kerr nor Dr. Allvey had ever heard of the illness or name of Ann Foulkes. My meeting with them both, on the same day, was a matter of much surprise. 'Tis true, I seized the rare opportunity of taking two such experienced physicians, and the surgeons of the Bedford Infirmary, to see A. F. to examine the state of her health, and to hear her story from her own mouth. The result of their visit is well known. I regret that these apparent trifles should compel me to intrude so much upon your attention; but the inuendoes of Dr. Y. must be explained, and the conduct of gentlemen of the highest respectability placed in a proper point of view.

I may likewise repeat, by positive assertion, that I thought it impossible Dr. Y. could have misunderstood the original difference of opinion, and the meaning of the written document, so much as to imagine that the symptom of vomiting of urine was for a moment thought of unconnected with the peculiarities of A. F.'s case.

It serves the purpose of Dr. Y. to separate the abstract question question from the case; but I must inform him that in so doing he acts towards others and myself in a manner that cannot but be highly disapproved of, by every candid and unprejudiced mind.

His assertion, that the impropriety of holding a medical meeting in his absence was hinted to me, I must beg permission to deny for the present, having no recollection of such a hint being given. All the medical men present fully agreed upon the propriety of informing Dr. Y. of our intention of seeing A. F., and of requesting his attendance likewise. A message was sent to his house, but he was from home, and not expected to return before the evening. It was out of the question, in this case, for two physicians coming from so great a distance to await his arrival. Dr. Y. never, I believe, thought it necessary to consult with a single practitioner of the county, upon this curious case, which he and the druggist had the entire management of. Why did he not deprecate the idea of a druggist prescribing for a woman laboring under such a supposed marvellous disease; the history of which he meant to lay before the medical world, which he was at that time taking notes of, and which was altogether under his express management? If not, why was the druggist compelled to send a copy of all his prescriptions to Dr. Y. previously to administering his medicines? How complimentary it is to all other medical men, for Dr. Y. to declare that they be of no service, because he was puzzled; and how strange it was, that it never struck him there might be some danger of the druggist doing incalculable mischief, by giving his emetics and diuretics, in this supposed case of Meteorismus Ventriculi! It is, of course, quite new to me, that other medical gentlemen thought with Dr. Y. upon the subject of the distension of the stomach *when they examined it;* nor do I think that Sauvages would allow it to be compatible for a weakly woman to labor under the Meteorismus Ventriculi, whilst she could talk for upwards of half an hour with as much strength of lungs as most robust men are blessed with.

If Dr. Y. cannot reconcile the apparent state of general good health, which I speak of, with the sufferings he frequently witnessed in A. F. I must refer him to those medical gentlemen whose names are enumerated in a note attached to the very able communication of Mr. Pulley, in your Journal for the present month. Every one must acknowledge the extreme difficulty of detecting a well-planned system of imposture. Witness the recent case of Ann Moore, of Tutbury: and, as it is within the recollection of most of the present medical officers of Guy's Hospital, I will direct the the attention of your readers to an extraordinary case which occurred there a few years ago, in the person of a woman, who, after a long watching, was detected in drinking her urine during the night, in order that she might not be supposed to have it in her power to evacuate her bladder.

It would have been better for Dr. Y. not to have made the unfortunate observations he does respecting the introduction of the catheter, seeing that A. F. and her mother declare he asserted it might be the death of her, if the bladder was examined by that instrument.

Not for a moment do I allow that the very true tadpole story can, by the most forced construction of any thing in my paper, be made to reflect upon the opinions of those celebrated men whose names Dr. Y. enumerates, and I most highly venerate.

Dr. Y. cannot now be offended if I say,

Mutato nomine, de te
Fabula narratur.

It is the case alone, I beg leave to repeat, and not the physiological question, to which I always consider myself bound to attend, and which, Dr. Y. well knows, he should have proved or disproved for the satisfaction of the public, before he wandered into such a long history of marvellous statements respecting ischuria and vomiting of urine. I cannot but admire his ingenuity in trying to get rid of this case with so much nonchalance: does he not remember telling me that he never lost sight of it, because he was of opinion that the disease which existed about the stomach, from the collection of urine, must soon kill A. F.; and that he had obtained a promise of being permitted to inspect her body after her decease? Dr. Y.'s own assertions, and the repeated declarations of A. F., would make it appear that he paid more attention to this affair, during the early stages of the urinous vomiting, than he is now ready to acknowledge.

Whether my paper was a more angry tirade than causes justified me in making it, the public must decide:—whether my account of the voluntary muscular efforts to blow out the epigastric region, in the manner which A. F. did, and which any person can imitate, is so confused as Dr. Y. represents it, I must not determine; but my friends think differently from him:—and whether he thinks A. F. is or is not an impostor, is what I am still extremely anxious to be informed of. I am, Sir,

Your very obedient Servant,

*Bedford, Dec.* 12, 1813. JAMES GIBBON.

